# Design of a Water Environment Monitoring System Based on Wireless Sensor Networks

**DOI:** 10.3390/s90806411

**Published:** 2009-08-19

**Authors:** Peng Jiang, Hongbo Xia, Zhiye He, Zheming Wang

**Affiliations:** 1 Institute of Information and Control, Hangzhou Dianzi University, 310018, Zhejiang Province, China; E-mails: sink@stu.hdu.edu.cn (H.X.), endyriver@tom.com (Z.H.); 2 Environmental Science Research & Design Institute of Zhejiang Province, 310007, China; E-Mail: wzhem@sohu.com (Z.W.)

**Keywords:** water environment monitoring, wireless sensor networks, data monitoring nodes, data base station, remote monitoring center

## Abstract

A water environmental monitoring system based on a wireless sensor network is proposed. It consists of three parts: data monitoring nodes, data base station and remote monitoring center. This system is suitable for the complex and large-scale water environment monitoring, such as for reservoirs, lakes, rivers, swamps, and shallow or deep groundwaters. This paper is devoted to the explanation and illustration for our new water environment monitoring system design. The system had successfully accomplished the online auto-monitoring of the water temperature and pH value environment of an artificial lake. The system's measurement capacity ranges from 0 to 80 °C for water temperature, with an accuracy of ±0.5 °C; from 0 to 14 on pH value, with an accuracy of ±0.05 pH units. Sensors applicable to different water quality scenarios should be installed at the nodes to meet the monitoring demands for a variety of water environments and to obtain different parameters. The monitoring system thus promises broad applicability prospects.

## 1. Introduction

The water environment, consisting of the surface water environment and underground water environment, can be differentiated to water bodies like rivers, lakes, reservoirs, oceans, swamps, glaciers, springs, and shallow or deep underground waters. The water environment, as well as other environmental elements like soil, organism and atmosphere, etc, constitute an organic complex. Once a change or damage to the water environment is observed in this complex, changes to other environmental elements inevitably occurs [1]. Due to the speed of China’s economic development, we can also see the resulting speeding-up of contamination and damage to the water environment. In this sense, water environment monitoring, as one of the major methods for water resource management and water contamination control, is found to be more and more indispensable.

At present there are mainly four methods for monitoring water environments, each of which has its advantages and disadvantages:

1. Artificial sampling with portable water quality detecting devices and subsequent lab analysis. This method applies only to samplings on cross-sections of river and lakes with a sampling frequency ranging from several times a day to monthly.
2. Automatic and continuous monitoring of water environment parameters by an automatic monitoring system consisting of monitors & control centers, as well as several monitoring sub-stations. Data can be remotely and automatically transferred. Each station provides its real-time water environment parameters. These systems can be costly and have a great influence on the surrounding ecological environment.
3. Water environment monitoring with remote sensing technology, namely detecting the spectrum specifics of an electromagnetic wave (radiation, reflection and scattering) in a non-contacting method with respect to the water body. After the processing of the information from the collection of illustrative spectra, its physics and chemical characteristics are to be identified. However this method can only provide a low accuracy, and it is also hard to perform real-time monitoring.
4. Water quality monitoring technology realized using some sensitivity of aquatic organisms to the presence of poisonous substances in water bodies by measuring or analyzing the change of activities of different organisms in different water environments, then coming to a qualitative evaluation report of the water quality. Basic measuring methods of this type being practiced include Fish Measuring and Beach Louse Measuring. Still, these methods can by no means be expected to reach high accuracy for water environment monitoring.

It is obvious that in a country like China, which has such an enormous water area, so diverse water bodies, so scattered spots on a water monitoring network, it will be insufficient to rely on the present numbers of monitoring stations and traditional monitoring technologies to satisfy the current monitoring needs, which emphasizes the fact that water environment monitoring must be continuous, dynamic, macro-scale, and swift; the water quality forecast must be prompt and accurate. In this sense, research and development on dynamic water environment monitoring technology, meeting the above-mentioned needs, must be conducted urgently, in order to achieve accuracy and comprehensiveness in reports of the changing situation of the water environment and finally reduce water contamination.

Compared with the present water detecting methods, constructing a monitoring system based on the WSNs (wireless sensor networks) would present us with several advantages such as low cost, convenient monitoring arrangements, collection of a variety of parameters, high detection accuracy and high accountability of the monitoring network, etc.

A WSN (wireless sensor network) is an *ad-hoc* network system composed of a great number of tiny low cost and low power consumption sensing nodes which are capable of sensing, calculating and communicating data [2]. It is also an intelligent system, which automatically accomplishes all types of monitoring tasks in accordance with the changing environment. Typical real-time water environment monitoring systems of based on WSNs found abroad are systems such as EMNET (by Heliosware, USA), Fleck (by CSIRO, Australia), LakeNet (by Notre Dame University, USA) and SmartCoast, designed by researchers from Ireland [3–7]. China has also been conducting research on the essential technology for real-time monitoring systems for water environments based on WSNs [8,9].

This paper studies and develops a water environment monitoring system based on a WSN, which was applied to water monitoring in an artificial lake, to realize remote and automatic on-line monitoring of both the pH and temperature of the lake water. The second part of the paper describes the comprehensive structural design of the monitoring system. The third part more specifically discusses the design of hardware and software of the data monitoring nodes. The fourth part explains the design of hardware and software of the data base station. The fifth part describes the software design for the remote monitoring center. The sixth part analyzes how this system is applied in pH monitoring in an artificial lake. The seventh part presents a summary of paper.

## 2. Design of Monitoring System

**Figure 1.**
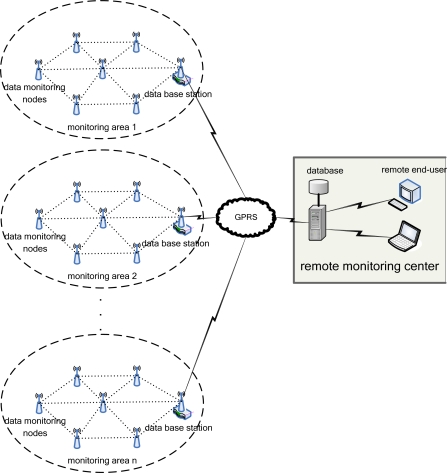
Water environment monitoring system based on WSNs.

The proposed water environment monitoring system based on a WSN is illustrated in Figure 1. It can be divided into three parts: data monitoring nodes, data base station and remote monitoring center for the water area being detected [9]. A great number of data monitoring nodes, distributed in water area to be detected, dynamically constitute a monitoring network, in which each node can only collect parameters such as pH, amount of dissolved oxygen, electrical conductivity rate and temperature, but also is capable of operating linearization and temperature compensation, data packaging, collected parameter memorizing and routing to a data base station; the data from the monitoring nodes is transferred to a remote monitoring center by the base station via a GPRS network; the monitoring center analyzes and processes the water quality parameters, gives an alarm for emergencies like water contamination, in addition any sudden changes in water quality, and provides support for decision-making in prevention and remediation of water contamination; the end-user can also realize an all-weather detection on the target water area via the Internet. The whole water environment monitoring system presents useful characteristics as large network capacity, flexible disposition, low power consumption, low cost, and minor influence on the natural environment.

## 3. Design of Hardware and Software for the Data Monitoring Nodes

Monitoring nodes, as the basic unit of the monitoring area of the water environment, are the basic units to realize the monitoring function. At present, the major functions of the monitoring nodes are:

1. Collecting the temperature and pH data of the water area to be monitored. The pH and temperature sensors installed on the monitoring nodes can meet the above needs and realize the linearization and temperature compensation for the data collected.
2. Setting up a wireless network based on the ZigBee protocol. A great number of monitoring nodes and base station a dynamically assembled into a wireless network based on the ZigBee protocol, via which the pH and temperature data, etc., shall be routed to a base station.

**Figure 2.**
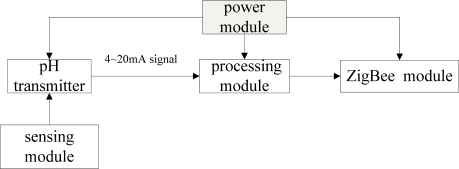
The system architecture of a data monitoring node.

Figure 2 shows the systematic structure of the monitoring nodes, which can be divided into five modules: processing module, pH transmitter, sensing module, power module and ZigBee radio frequency module. The mentioned modules, except for the sensing module, are unexceptionally placed in a water-proof cabin floating on the water surface with a dropped anchor. The sensing module is in the water, connected to the water-proof cabin via cable. All five modules have gone through a water-proofing process. The power module provides electricity for the pH transmitter, processing module and ZigBee radio frequency module. The pH transmitter collects the pH and temperature in the target water area after being connected to the pH and temperature sensors. The transmitter can convert the pH and temperature signals to a standard 4–20 mA signal. The processing module processes and memorizes the pH and temperature signals and collects the standard signals, then transfers them via the ZigBee module to the base station. Each node is connected with and controlled by the base station by the ZigBee communication protocol.

### 3.1. Design of Hardware for the Data Monitoring Node

#### 3.1.1. Design of the Transmitter

The pH transmitter is a LE-438 integrated pH and temperature sensor manufactured by METTLER TOLEDO. A weak voltage signal, output by the sensor, was converted to a standard 4–20 mA signal via the pH and temperature transmitter circuit.

**Figure 3.**
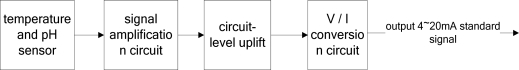
Transmitter module.

The transmitter circuit can be divided into two parts: the signal amplifying circuit and electrical level raising circuit. One magnifying circuit can amplify four-fold the original voltage from the pH transmitter. Since the original one is a two-way differential signal, it is still a two-way voltage signal after being amplified (−1.5 V to 1.5 V). The amplified signal should have its electrical level raised to 0–3.0 V to simplify the AD sampling of the microprocessor. Only the temperature signal should be amplified. The amplified voltage signal should convert the 0–3 V voltage signal to a standard 4–20 mA signal through V/I conversion circuit and output to an AD module in the MCU. Figure 3 shows the transmitter.

In the designing the transmitter, how much the signal shall be amplified and how high the electrical level shall be raised can all be adjusted according to the practical needs. The output signal of several types of sensors can be switched to the 0–3 V range and 4–20 mA standard electric current output as signals. This flexible design enables the monitoring nodes to adapt to all kinds of sensors with different settings.

#### 3.1.2. Design of the Processing Module

The MCU in the processing module is the MSP430F1611, manufactured by Texas Instruments. The MSP430F1611 is a type of MCU with low power consumption, which makes it extremely suitable for the power consumption design requirement. The inside of the MSP430F1611 has integrated 2-channel and 12-digit A/D converter, which can realize the AD conversion of the 4–20 mA standard signal from the transmitter.

**Figure 4.**
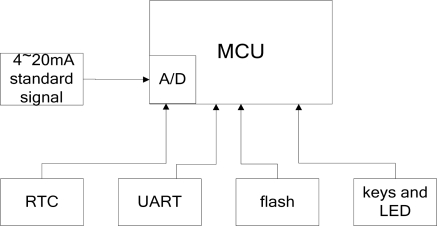
Processor module.

Figure 4 shows the node processing module, which includes the MCU module, real-time clock module, UART module, flash module, keyboards and LED module. The 4–20 mA signal, converted from the pH signal and temperature signal, will be input into the AD module integrated in the MUC to realize the AD conversion. After that, MCU will store the pH and temperature parameters according to the time sequence when they were collected. Finally, the MCU microprocessor will be communicating and organizing the network via the ZigBee module. In the meantime, the MCU microprocessor is connected separately with a real-time clock module, UART module, flash module, keyboard and LED module to realized functions such as time reading and writing, RS-232 SLIP communication, data storage and historic data reading, as well as man-machine communication.

#### 3.1.3. Design of the Interface Circuit for the ZigBee Radio Frequency Module

**Figure 5.**
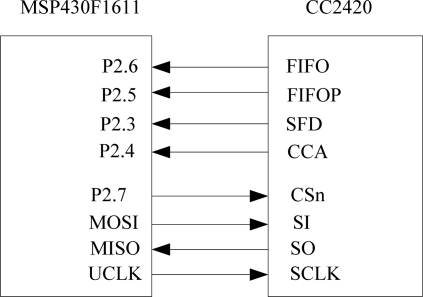
Interface circuit of the ZigBee module.

A CC2420 radio frequency receiving and sending chip has been selected as the chip of ZigBee module. CC2420 is an industrial class radio frequency receiving and sending chip developed by Chipcon. With a few peripheral components, this chip can receive and send data reliably in the wireless working frequency ban between 2.400 GHz–2.4835 GHz. The interface of the CC2420 includes SFD, FIFO, FIFOP, CCA and SPI (CSn, SI, SO and SCLK). By controlling the state of the pins of the FIFO and FIFOP, we can set up the Tx/Rxtemporary register; by setting up the state of the pins of the CCA, we can clear channel and by setting up the state of the pins of the SFD, we can control clock and time information input. Through the SPI bus, the CC2430 can set the operating mode of the chip, read and write the buffer data, and operate the status register. The interface circuit of the ZigBee module is shown in Figure 5.

#### 3.1.4. Design of the Power Module

**Figure 6.**
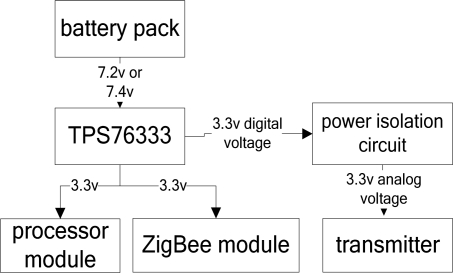
Power module.

The designed input power of the monitoring node is a voltage of 5–9 V. In the preliminary tests, we can choose the corresponding switch as the power supply. If batteries are needed for providing power, six nickel-hydrogen batteries (7.2 V) or two lithium batteries (7.4 V) can be used as power supply. Besides, the transmitter and motherboard can share the same batteries. The input voltage can be adjusted to a digital power output of +3.3 V through the TPS76333 chip and provide +3.3 V analog voltages after isolated processing. The power module is illustrated in Figure 6.

### 3.2. Design of Software for the Data Monitoring Node

The development environment for the system software is IAR Embedded Workbench for MSP430, and the programming language is C [10]. The system software can be divided into two modules: the main processor program, which is responsible for processing the water environment parameters collected by the sensors, and the ZigBee wireless communication program, which is designed for receiving and sending the water environment parameters. The integration of the two modules enables the nodes to sense, collect, process and transfer the water parameters.

#### 3.2.1. Design of the Master Routine

**Figure 7.**
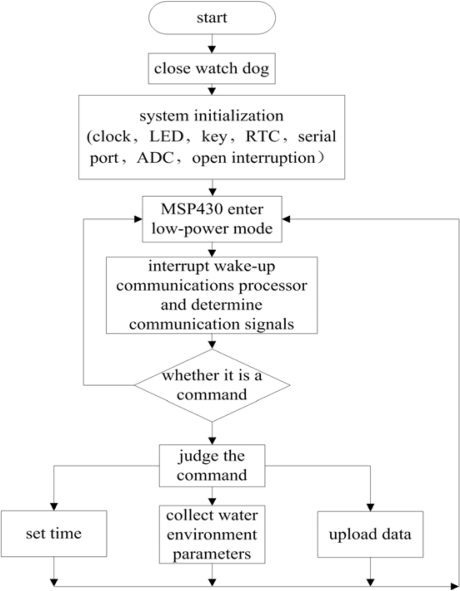
The flow diagram of the master routine.

As the main controller of the whole system, the MSP430F1611’s major responsibilities are initializing the system, receiving and executing the orders and memorizing the water parameters. The flow of the main programs is illustrated in Figure 7.

The operation of the main program of processor can be divided into five parts: (1) Setup the system, including initializing the clock, LED, KEY, RTC, Serial Port, ADC; setup the ZigBee module and switch off. (2) Processor goes into low-power-consumption mode and waits for the switch-off from the serial port. (3) The data input at the serial port will interrogate its breaking off and wake up the processor to resume normal working; it can also identify and operate the data at the serial port. (4) Decide whether the data received at the serial port is useful. If not, the processor shall return the low-power-consumption mode and keep on waiting for the serial port data; if useful, the processor shall decode and identify them and decide the content of the order. (5) As per the content of the order, by controlling the peripheral equipment, the processor sets up the time, measures the water parameters or uploads water quality parameters at a certain time. After the operation, the processor returns to the low power-consumption mode and waits for the data from the serial port.

#### 3.2.2. Design of ZigBee Wireless Communication Program

Table 1 shows the format of the data frame of the CC2420, which includes Lead Frame, Start Frame, Length Frame, Control Frame, serial number, address information, transferring data, RSSI and CRC [11]. Among the above-mentioned, the first 7 digits of the Length Frame define the length of the data series; the 8th digit should be reserved so that the data series can support 128 bytes at best and any length short of 128 bytes should be complemented with a 0.

**Figure 8.**
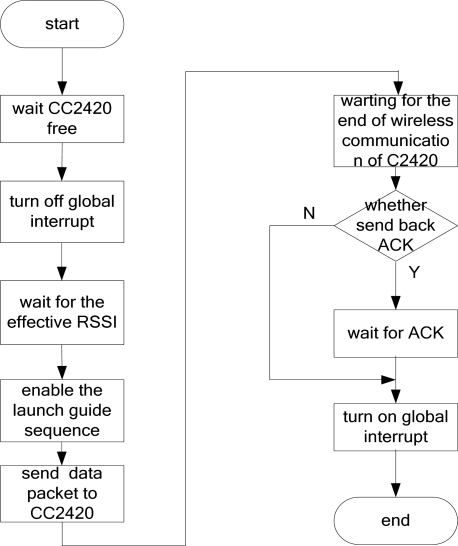
The flow diagram of the CC2420 send process.

Figure 8 is the flow chart for the sending program of the CC2420. After the start-up of the sending program, we need first detect whether CC2420 is free because only when it is free can data be sent. When the CC2420 is free, the program is shut down and the whole operation is in an interrupted state. After that, the sending program should wait for RSSI till it is effective and enable the launch guide sequence of the CC2420. Then the data packet will be read in the CC2420. Here, CC2420 should start up the radio frequency sending module and send the data via 2.4 GHz frequency band. At the completion of data sending by the CC2420, the program will decide whether the data should be send back by ACK. If there is such a need then wait, or just jump this step. Later the program shall start up the overall interruption and exit the sending program.

**Figure 9.**
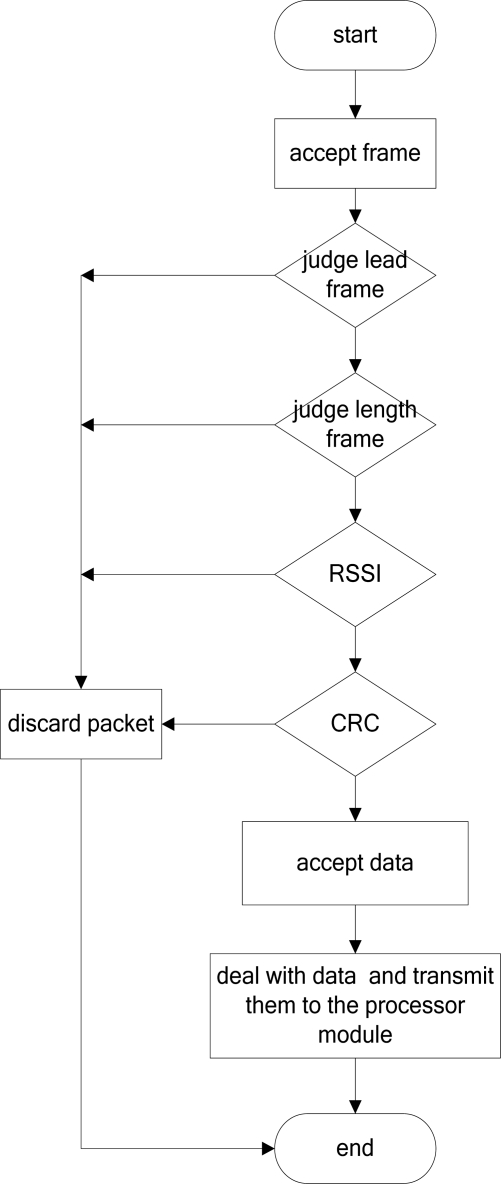
The flow diagram of the CC2420 accept process.

Figure 9 illustrates the CC2420 sending program. When receiving the data packet, the program should decide if the lead frame is correct. If incorrect, the program should discard the packet and end the receiving process. If the first data frame was correct the program should check its length. The program will also discard the packet if the frame is of the wrong length. Till the frame was checked at the correct length, the program later decides if the RSSI checkout and CRC checkout are correct. If they are incorrect, the program should again discard the data packet and end the receiving process. If the checkouts are correct, the program should read in the data in the packet and convert the format of the data, transfer to processor module and end the receiving process.

## 4. Design of Hardware and Software for the Data Base Station

**Figure 10.**
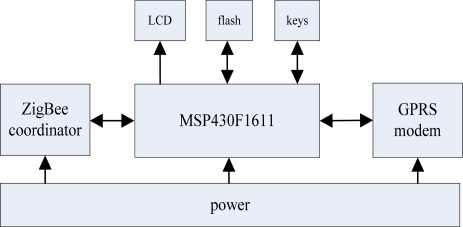
System hardware block diagram.

The hardware of the data base station uses a MSP430F1611 as the main processor to control the data base station; CC2430 is used as a co-processor to transmit monitoring data based on the ZigBee protocol between the data base station and data monitoring sub-network; a GPRS module is used to realize remote data communication between the data monitoring center and data base station; An AT45DB081D is used as the system’s solid memory to store historical data, and the buttons and LCD are supplemented as a man-machine interface. The system hardware block diagram is shown in Figure 10. The software of the data base station uses μC/OS-II embedded operating system as the software platform of the MSP430F1611 to improve the real-time performance of the system; a ZigBee 2004 stack from Chengdu Wireless Dragon Information Technology Company is used as the software platform of the CC2430 module.

### 4.1. Design of Hardware for the Database Station

#### 4.1.1. Design of the Interface Circuit for the ZigBee Module

**Figure 11.**
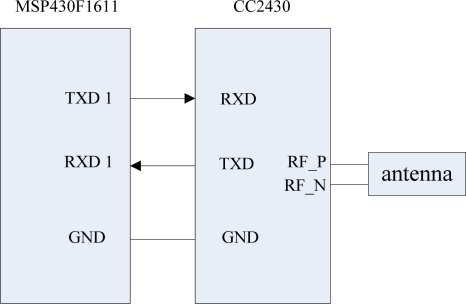
Interface circuit of the ZigBee module.

The ZigBee module design uses the a CC2430 module from Chengdu Wireless Dragon Technology. The CC2430 contains 8 K SRAM and 64 K Flash memory, so it is no longer needs to increase memory. The UART 1 interface circuit which is used to connect the ZigBee module and MSP430F1611 is shown in Figure 11. The CC2430 connects with the MSP430 through the serial port. In order to ensure that the two modules have a same voltage reference, a ground is needed to connect the two modules.

#### 4.1.2. Design of the Interface Circuit for the GPRS Module

**Figure 12.**
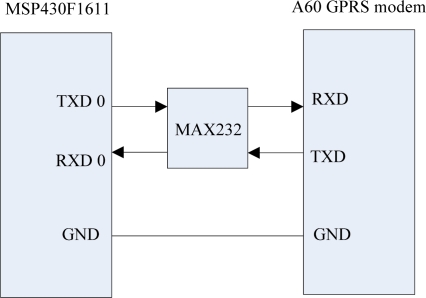
Interface circuit of the GPRS module.

GPRS is an acronym for General Packet Radio Service, and it provides medium speed data transmission [12]. GPRS connects with the MSP430F1611 through the serial port, and the MSP430F1611 sends AT commands to the GPRS Modem to control its data transmission. The instantaneous peak current of the GPRS module is about 2 A when the GPRS Modem is starting to connect with the GPRS network, so the power module must to provide a current of more than 2 A for the GPRS module. Also, a ground is needed to connect between the GPRS module and the MSP430F1611. The UART 0 interface circuit which connects the GPRS module and the MSP430F1611 is shown in Figure 12.

#### 4.1.3. Design of the Power Module

**Figure 13.**
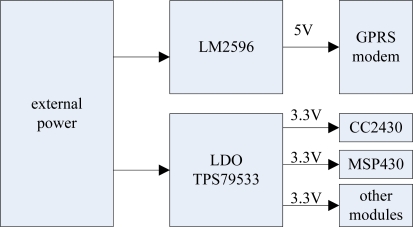
Power module.

A good power module is the foundation of a reliable system. Because of the high peak current of the GPRS module and the strong interference with the other modules when the GPRS module is in wireless communication, the design of the power module must include high power isolated chips in order to reduce this interference. The design of the power module is shown in Figure 13. It uses LM2596 and TPS79533 power chips. LM2596 is a power management integrated circuit, and its largest output current is 3 A. At the same time it has a good linearity and load regulation characteristics. TPS79533 is a single-output LDO with a fixed voltage (3.3 V). Since the data base station works outdoors, an external power supply can be a battery pack with 6 Ni-MH batteries (7.2 V) or two lithium batteries (7.4 V).

#### 4.1.4. Design of Other Modules

The LCD module uses the LCM19264 LCD which is a 192 × 64 pixel monochrome LCD. The MSP430F1611 does not open its bus, so it connects with the LCM19264 LCD by a general-purpose IO port. The data base uses AT45DB081D as its solid memory, and the AT45DB081D connects with the processor through the SPI interface. There is a JTAG circuit on it, either. Due to the small number of keys used, we use an independent keyboard to simplify the hardware design of the system.

### 4.2. Design of Software for the Data Base Station

This system is mainly designed using C language. During the transplantation of the μC/OS-II, we use assembler language to design the functions related with the hardware. As a result of the dual processor structure, the software design includes the CC2430 module’s software design and the MSP430 module’s software design.

#### 4.2.1. Design of Software for the CC2430 Module

**Figure 14.**
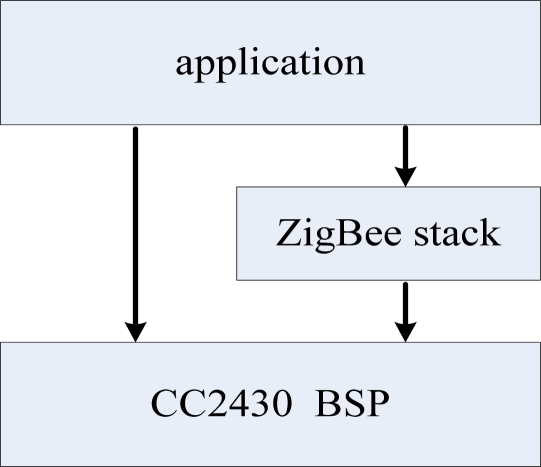
ZigBee module software level diagram.

The CC2430 module’s software consists of the ZigBee protocol stack, applications and board support package. The ZigBee software level diagram is shown in Figure 14.

The main purpose of using BSP in the CC2430 is to complete the package hardware, and provide a wide range of function calls to the upper ZigBee protocol stack and applications. The ZigBee protocol stack uses the Chengdu Wireless Dragon ZigBee 2004. The protocol stack is a lightweight protocol stack consisting of two parts: one is the IEEE802.15.4 definition of the physical layer and MAC layer protocol; others are the network layer, security layer and the application programming interface which are defined by the ZigBee Alliance. ZigBee protocol stack that defines three objects according its functions: network coordinator, routers, and semi-functional nodes [13]. To control the entire ZigBee network, the ZigBee module of the data base station should have the function of network coordinator.

**Figure 15.**
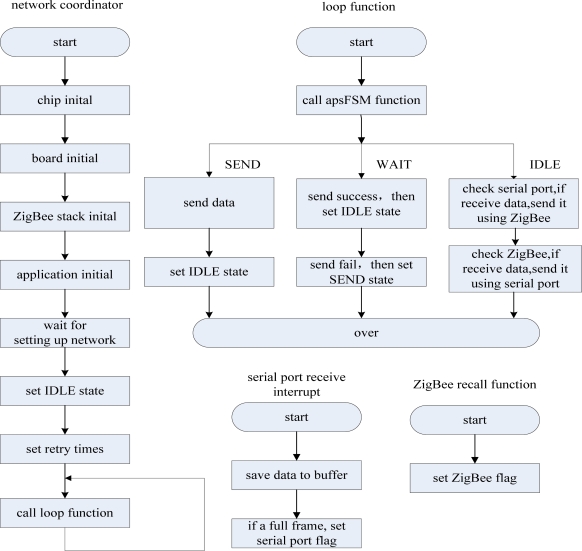
Application level process flow diagram.

The main mission of the CC2430 module’s application is to call the application programming interface provided by the ZigBee protocol stack and BSP to build and maintain a network, and undertake the data transfer mission between the MSP430 and ZigBee networks. When a node joins the network, the network will assign a network number to the node, and a message will be sent to the MSP430 module of the data base station to inform it to update its node state table. If the network lost a node, a message will be sent to the MSP430 to inform it to update its node status table, too. At the same time, it is responsible for monitoring the data base station and the data monitoring node. The CC2430 module does not deal with any data and it is only responsible for transmitting data. The application level process flow diagram is shown in Figure 15.

#### 4.2.2. Design of Software for the MSP430 Module

**Figure 16.**
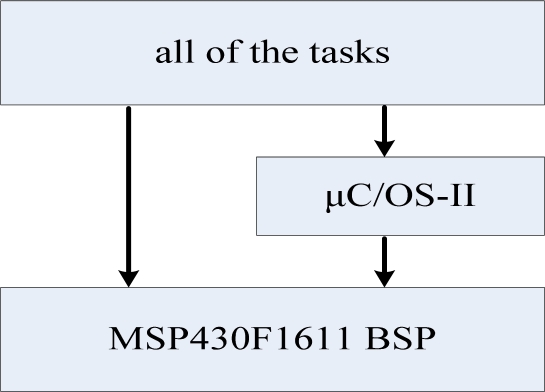
MSP430 module software level diagram.

In order to improve the reliability of the monitoring system, enhance the real-time performance and simplify the system’s programming, we transplanted the μC/OS-II embedded operating system to the MSP430. The MSP430 module software level diagram is shown in Figure 16.

The MSP430F1611 BSP mainly includes clock, serial port, DMA, timer and other hardware drivers. The development tool of the MSP430F1611 is IAR 3.42. The transplant of μC/OS-II requires two steps: the first one is to properly configure μC/OS-II, i.e., by correcting a number of compiler-related data types and macro definitions, and the other is to prepare a number of processor-related functions. The μC/OS-II requires an interrupt from time to time as its time tick interrupt. The time tick interrupt can be generated by the watchdog of the MSP430F1611 and the cycle of the interrupt can be set at 32 ms. The MSP430 module’s software consists of six tasks and the corresponding subroutines of the tasks. The main tasks are: key scanning task, LCD display task, ZigBee module communication task, GPRS module communications task, data process task, and flash task.

Because the data base station needs to respond to the commands from the data monitoring center in a timely manner, the GPRS module communicating task should be allocated the highest priority. At the same time, when the ZigBee module communicating task communicates with the monitoring node, the command which comes from the data monitoring center must be delivered to the data monitoring node in a timely way, so it should have the next highest priority. The data processing task has the third priority. The flash task has the fourth priority. The key scanning and LCD display tasks have the fifth and sixth priorities.

**Figure 17.**
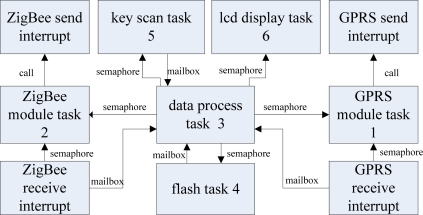
The communication diagram of each task.

The other tasks and interrupt service subroutine deliver the message and the keys to the data process task, and the data process task notifies the other tasks to deal with the message. The other tasks and interrupt service subroutine communicate with the data process task through a unified message mailbox and data process task activates other tasks by Semaphore. The communication diagram of each task and the corresponding interrupt service subroutine is shown in Figure 17.

##### (1) GPRS module communication task

**Figure 18.**
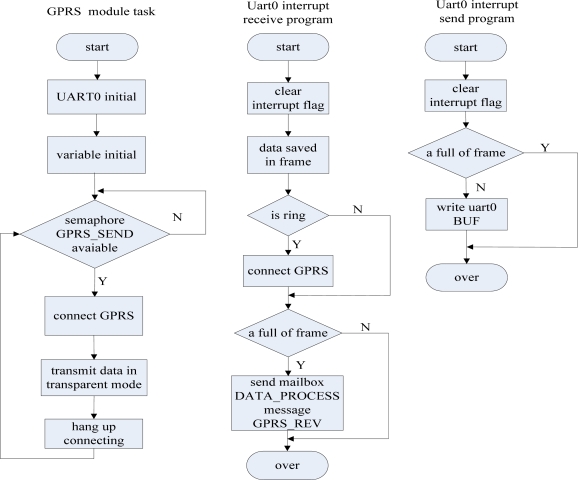
The flow chart of GPRS module communication task and the corresponding serial interrupt service subroutine.

The GPRS module’s communication task is responsible for setting up the GPRS network and communicating with the data monitoring center through this module. The GPRS module is driven by the MSP430 through its serial port and is controlled by AT commands. The data transmission of the GPRS task is transparent, and it is said that the GPRS module is only passive towards execution of the orders from the data monitoring center, so the GPRS module should be given an phone call to activate it. A serial receiver interrupt service subroutine detects the activation and sets up GPRS network after a phone call. If the GPRS module has no data to transmit for more than one minute, it will automatically enter the sleep state. Therefore, if the data base station does not receive a disconnection request with GPRS from the data monitoring center, you need to periodically (for example, every 50 seconds) send a heartbeat package to the data monitoring center to maintain the GPRS connection. The tasks and the corresponding GPRS serial port transmition and receiving interrupt service subroutine are shown in Figure 18.

##### (2) ZigBee module communication task

The ZigBee module communication task has the second highest priority. Through the task, the data base station sends the commands to the data monitoring nodes. That is, if the data process task sends a signal to the ZigBee task, the ZigBee task will be activated to send the corresponding command. When the serial port 1 receives a frame of data from a data monitoring node, it will send a message to the mailbox of the data process task to activate it to deal with the monitored data.

**Figure 19.**
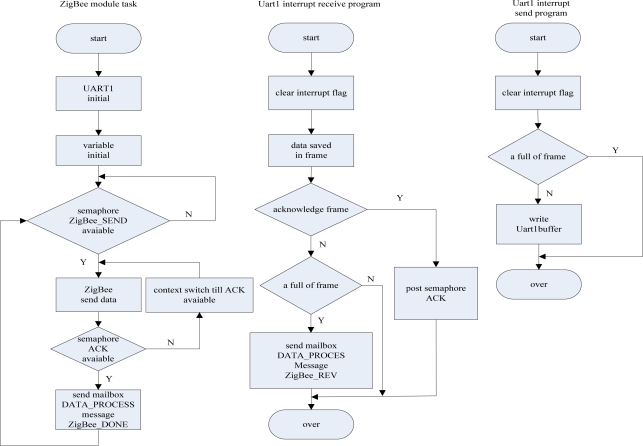
The flow chart of ZigBee module communication task and the corresponding serial interrupt service subroutine.

Because of the weakness of the ZigBee 2004 stack, the MSP430 and CC2430 use two wire serial ports for communication. When the CC2430 closes the global interruption, at the same time, if the MSP430 sends continuous data to the CC2430, it will be nothing to reflect the interruption and data will inevitably be lost. As the CC2430 uses a transparent transmission mode, in order to resolve this problem, we only have to use retransmission mechanisms. When the data base station sends a command to a monitor node, you must to wait for the confirmation information back from the monitor node before sending the next command, and when the data monitor node wants to send a message to the data base station, it also requires the confirmation information. Therefore, when the data base station receives some message from the data monitor node, the message will be checked by the data base station, and if the message passes the frame check, the data base station will give the node a confirmation frame, and if it cannot pass the frame check, the data base station will not send back the confirmation frame. After sending a frame of data, the node will stop sending data and waits for the confirmation frame from the data base station. If it is overdue, the node will re-send the message until the data base station sends back the confirmation frame. The overdue time is set to be 2 seconds. The task and the corresponding ZigBee serial port transceiver interrupt service subroutine flow chart are shown in Figure 19.

##### (3) Data process task

Data process task is mainly to analyze the command from the data monitoring center and the monitoring data from the data monitoring node, and activate the corresponding tasks to process these messages. It can be activated by the GPRS received interrupt subroutine and the ZigBee received interrupt subroutine through the mail box message. The commands from the data monitoring center can be divided into two categories: the first type is the commands to set up the data base station; the other type are the commands to control the data monitoring node. Among them, the commands to control the monitoring node mainly have the function to set up the sampling channels of the monitoring node and read the current sampling value of these channels.

**Figure 20.**
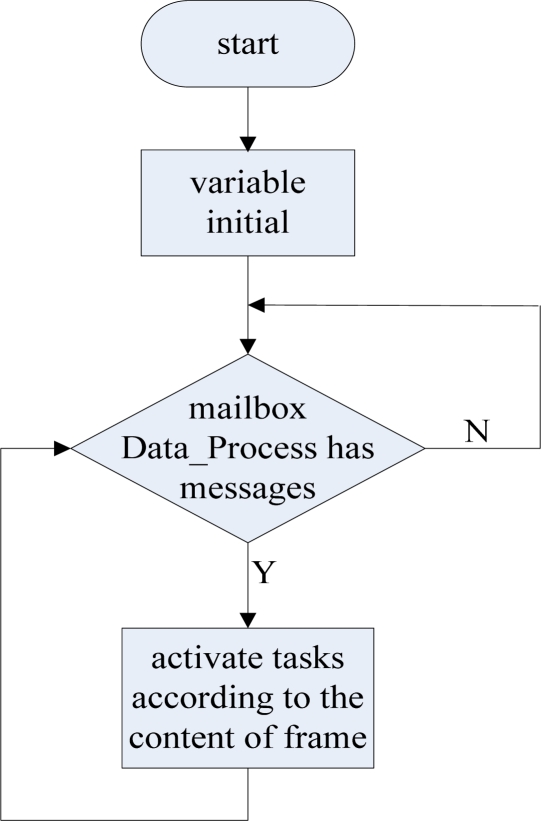
The flow chart of the data process task.

The data process task calls the corresponding module according to the command number in the command frame. If it is needed to maintain the GPRS connection, the data process task needs to send a heartbeat packet regularly to maintain the GPRS connection, and if there is no need to maintain the GPRS connection (that is, receive a GPRS disconnect request from the data monitoring center), we will stop sending heartbeat packets, then, one minute later, the GPRS module will automatically enter dormancy status. If the data process task receives a message from a new monitoring node, there is a need to update the node status table. The flow chart of the data process task is shown in Figure 20.

##### (4) Other tasks

The key scanning task, LCD display task and flash task will be activated by the data process task. The key scanning task is responsible for monitoring the keyboard, and if a key is depressed, it will activate the corresponding data process task to deal with it. The LCD display task mainly shows the status of the data base station and provides the man-machine interface. The flash task is responsible for reading and writing the flash memory.

## 5. Design of Software for the Remote Monitoring Center

The remote monitoring center consists of two parts: the GPRS gateway and the data center. The GPRS gateway is responsible for receiving the water environment parameters, and the data center connects with the GPRS gateway through the serial port. In addition, the remote monitoring center also includes the database and monitoring software. The monitoring software provides a complete monitoring interface to carry out historical data queries, display real-time data, data analysis and alarming for non-normal status. The remote monitoring center is responsible for monitoring the changes of the water environment, controlling and administering the on-site implementation, alarming for pollution emergencies and rapid environment changes in real-time.

## 6. System Performance

### 6.1. Time Synchronization

Time synchronization is an important performance in WSNs, because it is a key factor in the process called data fusion [14]. In this system, we make the base station the master clock of a monitoring area. A monitoring area is a cluster, and the node which is nearest from the master clock in the cluster will be selected to be time synchronized with the master clock. Other nodes near with this node will choose it as their synchronization source in the monitoring area, and the remaining nodes which are far away from the master clock will choose the nearest node as their synchronization sources. This method of time synchronization is similar to NTP [15,16]. Using a 25 Kb/s radio, 16 bits can be transmitted in 1 ms, so, if there are 100 nodes in a monitoring area, the largest time-delay between two nodes will less than 0.1 s. In this monitoring system, the smallest sampling frequency is 1 minute, which is much larger than the largest time-delay, so this system will work well with this kind of time synchronization.

### 6.2. Data Analysis

Since the monitoring system is a low-power systems, having a long sampling period which is often more than half an hour, and the data is stored on the local node, the system doesn’t have a lot of data traffic during the data sampling process. Only when the data monitoring center requests history data from the monitoring system, the nodes of the system will transmit their history data, which were stored locally to the base station, then the base station transmits all the date to the data monitoring center. Before the nodes store the monitoring data, they will perform the linearization and temperature compensation for the collected data. To summarize, we transmit all monitoring data stored on the nodes, but it is a passive transfer and it will not occur often. The communication pressure of the system is not so big, so we do not have to do to deal with data compression temporarily.

### 6.3. Reliability of Network Communication

In this monitoring system, we use *ad-hoc* multi-hop routing to support the the ZigBee wireless communication [17,18]. It uses a shortest-path-first algorithm with a single destination node and active two-way link estimation. The multi-hop router is essentially transparent to us and easy to transplant into our system. With the frame check and re-send mechanisms, we could make sure that all the commands and the monitoring data will be sent to the target device successfully. To sum up, our monitoring system has good performance in the wireless communication reliability area.

## 7. System Testing

**Figure 21.**
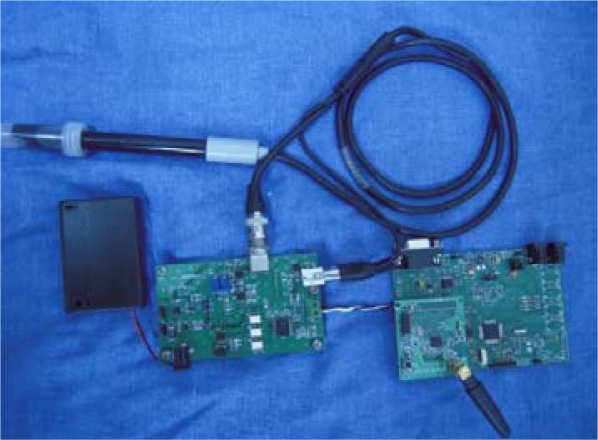
Actual data monitoring node.

From early November to the end of November in 2008, we tested the water environmental monitoring system we had designed in the artificial lake at HangZhou DianZi University. The data monitoring node, which uses the LE-438 pH and temperature sensor, is shown in Figure 21.

**Figure 22.**
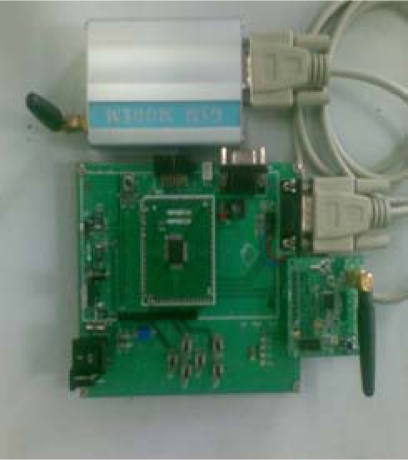
Actual data base station.

Its temperature measurement range is 0–80 °C, and the accuracy is ±0.5 °C; its pH measurement range is 0–14, and the accuracy is ±0.05. The actual data base station is shown in Figure 22.

The GPRS modem (on the top) is connected to the motherboard through the serial line. The CC2430 module is inserted at the right corner of the motherboard, and the MSP430 minimum system board is in the middle of the motherboard.

In this experiment, we deployed five nodes on the artificial lake to monitor the pH in real-time and online. During this experiment, the temperature was between 0 and 20 °C, and the rainfall was moderate. We observed changes in the pH range in the artificial lake from between 7.9 to 8.4, the max. value appeared between 3 PM–5 PM, and the minimum value appeared between 8 PM–9 PM. In addition, pH was also affected by rainfall. Before the test experiments, we calibrated the pH probe with standards of pH 4.0, pH 7.0 and pH 10.0.

**Figure 23.**
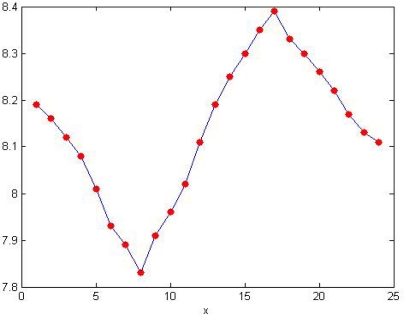
Curve of daily pH changes monitored by the system.

**Figure 24.**
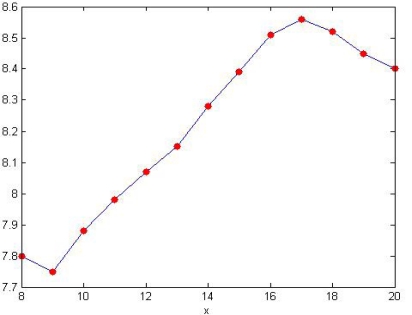
Curve of weekly pH changes monitored by the system.

The monitoring system automatically collects the temperature and pH of the water once every other hour. After compensation the pH and the temperature values collected by the five nodes should be separately transferred to base station via their own ZigBee communication module. The average should be uploaded to the remote monitoring center. Figure 23 is the daily changing curve of pH. Figure 24 is the weekly changing curve.

It is perceptible in Figure 23 that the maximum pH value occurs between 3 PM–5 PM. This is because the phytoplankton in the artificial lake comes to its peak photosynthesis. Below are the chemical equations of the photosynthesis to explain the daily changing rule of the pH:

$$\begin{array}{c} 6\;{\text{CO}}_{2}+12\;H_{2}O=>C_{6}H_{12}O_{6}+6\;H_{2}O+6\;O_{2}\\ {\text{CO}}_{2}+H_{2}O=>H_{2}{\text{CO}}_{3} \end{array}$$

During the peak time of the photosynthesis plenty of CO_2_ had been changed to C_6_H_12_O_6_, which lowers the content of H_2_CO_3_ and so the acidity and raises the pH.

From the change of pH in Figure 24 we can see that the pH is influenced by the change of temperature and precipitation in the week time and has been fluctuating within a certain range. But the changes each day were quite similar.

**Figure 25.**
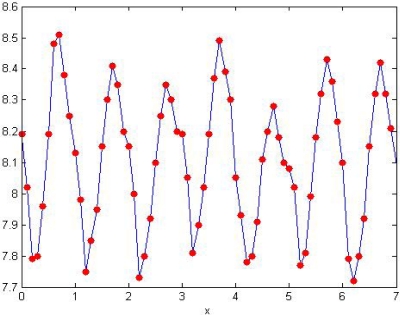
Curve of daily changing pH collected by artificial sampling.

During the experiment, the pH has been artificially sampled once every other hour. As shown in Table 2, by comparing the pH from artificial sampling and the monitoring system, the daily pH are quite similar, which indicates the accurateness and reliability of the pH detected by the monitoring system. Figure 25 illustrates the daily changing curve of pH collected by artificial sampling, which was done between 8 AM to 8PM once every other hour. The sampling process was labor intensive. The lab analysis of the sampling was far from prompt. In this experiment, coordinating with the monitoring system, the monitoring nodes designed by this experiment has shown to be accurate and efficient in the remote and real-time detection on the pH and temperature of the artificial lake.

## 8. Conclusions

A wireless sensor network was developed in the hope of tackling with the problem of the lack of a practical environment monitoring system. This monitoring system consists of three parts: data monitoring nodes, data base station and remote monitoring center. It presents us with useful features such as large monitoring ranges, flexible configuration, low power consumption, small damage to the natural environment and low cost.

This paper is devoted to the explanation and illustration for our new design of water environment monitoring system, based on a wireless sensor network. The system generally includes three parts: hardware and software of data monitoring nodes, hardware and software of the data base station, as well as software for the remote monitoring center. The system successfully performed an on line auto-monitoring of the water temperature and pH environment of an artificial lake. The system's measurement capacity ranges from 0 to 80 °C on water temperature, with an accuracy of ±0.5 °C and from 0 to 14 on pH value, with an accuracy of ±0.05. Sensors applicable to different water quality could be installed at the node to meet the monitoring demands in different water environments and to obtain different parameters. The monitoring system thus promises broad applicability.

## Figures and Tables

**Table 1.** Format of the data frame.

Frame name	Length(Byte)
Lead Frame	4Byte
Start Frame	1Byte
Length Frame	1Byte
Control Frame	2Byte
Serial number	1Byte
Address information	6Byte
Transferring data	nByte
RSSI	1Byte
CRC	1Byte

**Table 2.** Comparing the pH from artificial sampling and the monitoring system.

**Sampling time**	**AM 08**	**AM 09**	**AM 10**	**AM 11**	**AM 12**	**PM 01**	**PM 02**

**Artificial sampling**	7.80	7.75	7.88	7.98	8.07	8.15	8.28
**System sampling**	7.85	7.80	7.95	8.00	8.10	8.20	8.30

**Sampling time**	**PM 03**	**PM 04**	**PM 05**	**PM 06**	**PM 07**	**PM 08**	

**Artificial sampling**	8.39	8.51	8.56	8.52	8.45	8.40	
**System sampling**	8.40	8.55	8.60	8.55	8.50	8.45	
